# The NK1 receptor antagonist NKP608 inhibits proliferation of human colorectal cancer cells via Wnt signaling pathway

**DOI:** 10.1186/s40659-018-0163-x

**Published:** 2018-05-30

**Authors:** Xiao-Ling Niu, Jian-Feng Hou, Jing-Xiang Li

**Affiliations:** 1 Department of Traditional Chinese Medicine, Shanghai Pudong New Area Zhoupu Hospital, Shanghai, 201318 People’s Republic of China; 2 Department of Hepatobiliary Surgery, The First Hospital of Yulin City, Yulin, 719000 Shanxi People’s Republic of China; 3 Anorectal Department, Dongzhimen Hospital, Beijing University of Chinese Medicine, Hai Yun Cang on the 5th Zip, Dongcheng District, Beijing, 100700 People’s Republic of China

**Keywords:** NKP608, Colorectal cancer, Wnt signaling pathway, Proliferation

## Abstract

**Background:**

Neurokinin1 (NK1) receptor has played a vital role in the development of tumor. However, NKP608 as a NK1 receptor antagonist whether has the effect of the resistance of colorectal cancer is still unclear. Thereby, in this study, we investigated the role of NKP608 on human colorectal cancer and explored the underlying mechanism.

**Methods:**

The cell proliferation of colorectal cancer cells was detected by cell counting kit-8 (CCK8) assay, cell migration and invasion were assessed by transwell assay, the apoptotic ratio of cells was assessed by Annexin V-fluorescein isothiocyanate/propidium iodide stained and flow cytometry. The involvement of molecular mechanisms was examined by western blot.

**Results:**

In this study, we found that NKP608 inhibited the proliferation, migration/invasion of HCT116 cells. In addition, NKP608 reduced expressions of Wnt-3a, β-catenin, Cyclin D1, and (vascular endothelial growth factor) VEGF while induced expression of E-Cadherin. Furthermore, flow cytometry analyzed that NKP608 induced apoptosis of HCT116 cells, consistently, western blotting detecting of apoptosis-related proteins revealed that NKP608 downregulated Bcl-2 while upregulated Bax and Active-Caspase-3.

**Conclusions:**

Taken together, our results demonstrated that NKP608 inhibited colorectal cancer cell proliferation, migration and invasion via suppressing the Wnt/β-catenin signaling pathway. Therefore, NKP608 might represent a promising therapeutic agent in the treatment of colorectal cancer.

## Background

In China, the incidence rate of colorectal cancer rank as fifth as well as third in males and females, respectively, and colorectal cancer is the fifth commonly leading causes of cancer death among both sexes [1]. Risk factors for colorectal cancer encompass non-modifiable factors like age, race and ethnicity, genetic predisposition, several diseases and modifiable factors such as red meat, processed meat, obesity, smoking, alcohol consumption [2–6]. Chemotherapy as a chief means has improved survival rates, while it gives rise to a series of side effects. Therefore, more efficient treatments with unambiguous mechanisms are needed to be developed for colorectal carcinoma.

NKP608, a non-peptidic derivative of 4-aminopiperidine, emerges as a selective and specific substance P (SP) antagonist acting at neurokinin1 (NK1) receptors with anxiolytic or antidepressant effect both in vitro and in vivo [7, 8]. It has been reported that NK1 receptor antagonist such as aprepitant, L-733,060, L-732,138 exert an antitumor action in hepatoblastoma, small-cell- and non-small-cell lung, pancreatic, gastrointestinal cancer cells in vivo and in vitro [9–12]. Accumulating of evidence suggested that SP/NK1 receptor complex plays a vital role in tumorigenesis and development of carcinoma, based on SP implicating cancerous cell growth, neoangiogenesis and metastasis, further, NK1 receptors being overexpressed in tumoral cells and malignant tissues [13–15]. NK1 receptor antagonists, after binding to NK1 receptor, exerted an anticarcinogenic action including elicit inhibition of tumor cells proliferation, invasion, migration and induction of tumor cells apoptosis [16, 17], but therein molecular mechanisms involved are not elucidated.

It is established that mutation of Wnt/β-catenin exists in approximately 90% of colorectal tumors [18]. Moreover, Wnt signaling is believed to be of importance in regulation of cell growth, migration and differentiation, and some of studies have shown that aberrant activation of the Wnt/β-catenin signaling may play a crucial role in human tumourigenesis, including in colorectal cancer [19, 20]. Hence, the inhibition of Wnt signaling pathway is significant in the seek for potential antitumor drugs.

In the present study, we reported a novel inhibitor of human colorectal cancer cell, a NK1 receptor antagonist, NKP608. We present data showing, NKP608 reduced viability of HCT116 colorectal cancer cell lines by triggering apoptosis, possibly regulation of Wnt/β-catenin signaling pathway. Thus, our results demonstrated NKP608 could be a promising therapeutic compound for the development of effective anticancer therapeutics for colorectal cancer.

## Methods

### Chemicals and reagents

NKP608 was purchased from Medchem Express (New Jersey, USA). RPMI-1640 was supplied from Hyclone Laboratories (Logan, UT, USA). Fetal bovine serum (FBS) was from Gibco Cell Culture (Carlsbad, CA, USA). Cell counting kit-8 (CCK8) kit was obtained from Beijing Solarbio Science and Technology (Beijing, China). Metrigel was supplied with BD Biosciences (MA, USA). Annexin V-fluorescein isothiocyanate/propidium iodide (Annexin V-FITC/PI) Apoptosis Detection kit was from Beijing 4A Biotech (Beijing, China). Radioimmunoprecipitation assay (RIPA) lysis buffer and bicinchoninic acid (BCA) assay was from cwbiotech (Beijing, China).

Antibodies to Active-Caspase-3, Wnt-3a, β-catenin, E-Cadherin, (vascular endothelial growth factor) VEGF were obtained from Cell Signaling Technology, Inc. (Danvers, MA, USA). Other antibodies to Cyclin D1, Bcl-2, Bax, GAPDH and peroxidase conjugated secondary antibodies were provided from Proteintech Group, Inc. (Wuhan, China). The enhanced chemiluminescence (ECL) detection system was obtained from Proteintech Group, Inc. (Wuhan, China).

### Cell culture

Human colorectal cancer cell lines HCT116 and colonic epithelial cell line CCD841 were obtained from the American Type Culture Collections (Manassas, VA, USA) and were cultured at 37 °C containing 5% CO_2_ in RPMI-1640 medium supplemented with 10% FBS, penicillin G (100 U/ml), and streptomycin (100 μg/ml). Experiment was used with cells in logarithmic growth phase (0.5 × 10^6^–1 × 10^6^ cells/ml).

### Cell proliferation assay

Cell proliferative activity was evaluated using CCK8 following manufacturer’s instructions. After cultivation for 72 h, cells were treated by NKP608 with a series of gradient concentrations (0.001, 0.01, 0.1, 1, 10, 100 μM) or without NKP608 as control. 10 μl CCK8 solution was added to each well, then the growth of cells were assessed by measuring the absorbance [optical density (OD)] at 450 nm using a spectrophotometer (Thermo Fisher Scientific, MA, USA). According to the OD values corresponding to various concentrations, the following experiment concentration was identified. Cells were incubated with the NKP608 with the identified concentration and the negative control group (NC) with 1‰ DMSO in culture media for 24, 48, and 72 h, cells OD values were measured.

### Cell migration and invasion assay

Cells invasion and migration assay were performed using 24-well transwell chamber with or without Matrigel matrix in terms of the protocol provided by the manufacture. Briefly, after 24 h of NKP608 incubation, 100 μl serum-free medium suspension containing a total of 1 × 10^5^ cells were plated in the upper chamber and the lower chamber was filled with 500 μl of complete medium. After incubation for 24 h, residual cells were wiped with cotton-tipped swabs, invasive cells were fixed with 4% paraformaldehyde for 30 min and then stained with 0.1% of crystal violet for 20 min. Subsequently, the invaded cells were counted under a microscope in five random fields per well. For detecting cell migration, the steps were similar to detection of cells invasion, whereas, Matrigel was not applied.

### Detection of apoptosis

Apoptosis induction in HCT116 cells was measured using Annexin V-FITC/PI Apoptosis Kit according to the manufacturer’s instructions. After 24 h of NKP608 incubation, the HCT116 cells were digested using trypsin without EDTA, washed with ice-cold PBS, centrifuged at 1000 rpm for 5 min and then collected cell deposits. Added 1 × binding buffer, adjusted cells at a density of 1–5 × 10^6^ cells/ml and added 5 µl Annexin V-FITC and 10 µl PI. Apoptosis analysis was performed using flow cytometry.

### Western blotting analysis

Total cell protein was extracted with RIPA lysis buffer for 30 min and centrifuged at 12,000 rpm for 15  min to collect supernatants. The protein concentration was measured with a BCA assay, and then, samples were separated by 10% sodium dodecyl sulfate-PAGE (SDS-PAGE) and transferred onto poly vinylidene difluoride membranes. The membranes were then blocked with 5% nonfat milk in Tris-buffered saline buffer with Tween-20 for 1 h at room temperature and subsequently, incubated with primary antibody against Wnt-3a, β-catenin, E-Cadherin, VEGF, Cyclin D1, Bcl-2, Bax, Active-Caspase-3 (1:1000 dilution) and GAPDH (1:5000 dilution) in 4  °C overnight and then with horseradish peroxidase-conjugated anti-rabbit, anti-mouse secondary antibodies (1:5000 dilution). Finally, proteins were detected by ECL kit and western blot bands were measured with QUANTITY ONE software.

### Statistical analysis

Statistical analysis was conducted in SPSS 18.0 (SPSS, Chicago, IL, USA). All data were evaluated by an analysis of one-way analysis of variance and Student’s *t* test. Error bars are calculated standard deviation (SD) of the mean. Significance was considered as *p *< 0.05. In the graphs, asterisks on a column indicate statistical significance compared to the NC.

## Results

### NKP608 reduced HCT116 cells proliferation

**Fig. 1:**
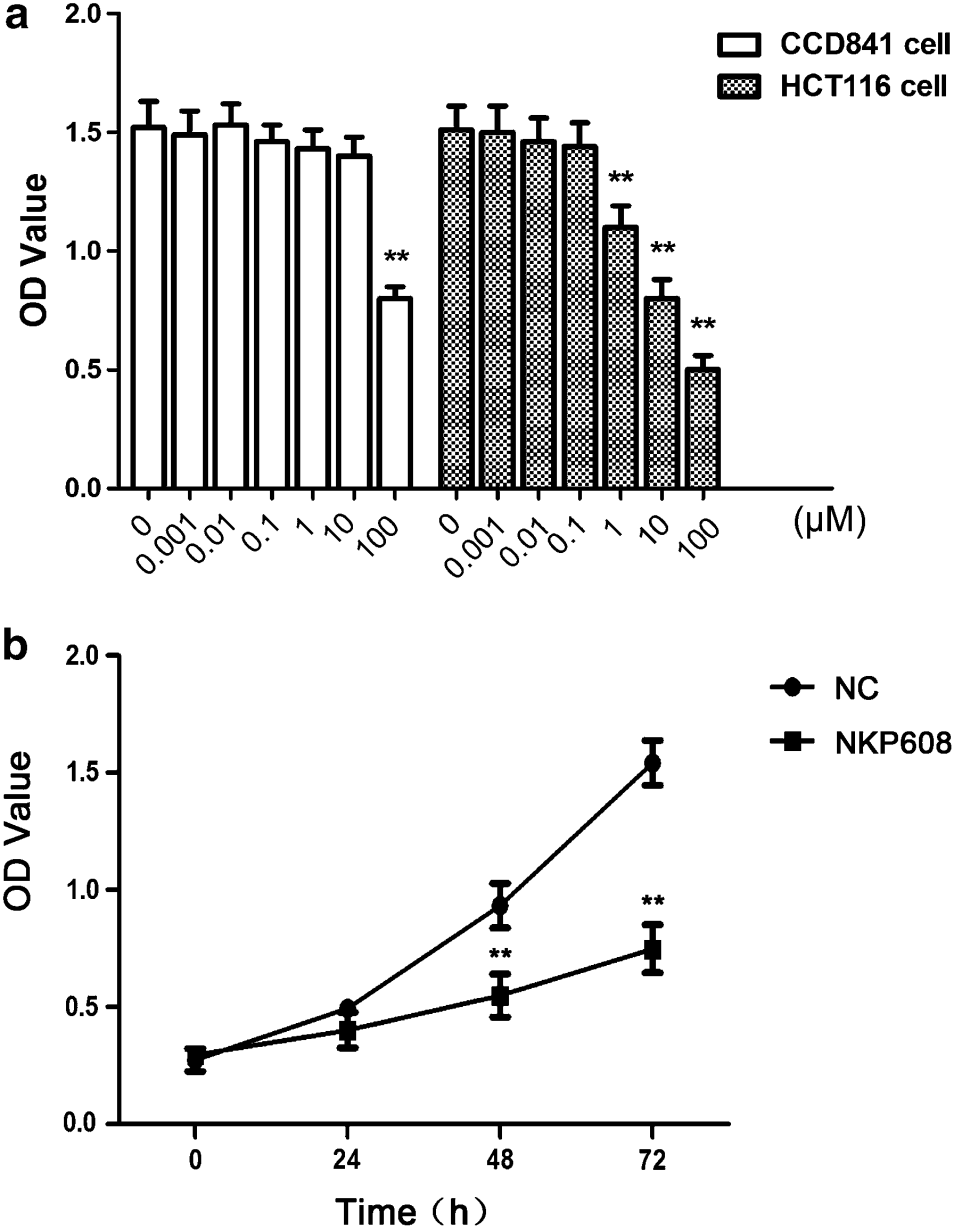
NKP608 inhibited HCT116 cells proliferation. **a** OD values of HCT116 cells were inhibited by NKP608 with a concentration-dependent manner while those of normal cells were not. **b** HCT116 cells were treated with NKP608 for 24, 48, 72 h and cell viability was quantified by CCK8 assay. Values are expressed as mean ± SD. *p < 0.05 versus without NKP608 groups. *p < 0.05 versus NC groups (with 1‰ DMSO)

In order to investigate the effect of NKP608 on BC, cell viability was measured by CCK8 assay. With the increase of drug concentration, the inhibition effect of NKP608 on BC cells was gradually increased (Fig. 1a), additionally, the viability of the normal BC cells was inhibited after treatment of NKP608 with 100 μM (Fig. 1b). Based on the dose-dependent change of OD value, the 10 μM was chosen as the following experiment concentration. Then, we treated HCT116 cells with 10 μM NKP608 for different time periods and then cell proliferation assay showed in Fig. 1b that NKP608 time-dependently decreased cell viability of HCT116 cells, especially cell viability at 48 and 72 h significantly decreased as compared to that at 0 h (*p < 0.05).

### NKP608 inhibited HCT116 cells migration and invasion

**Fig. 2:**
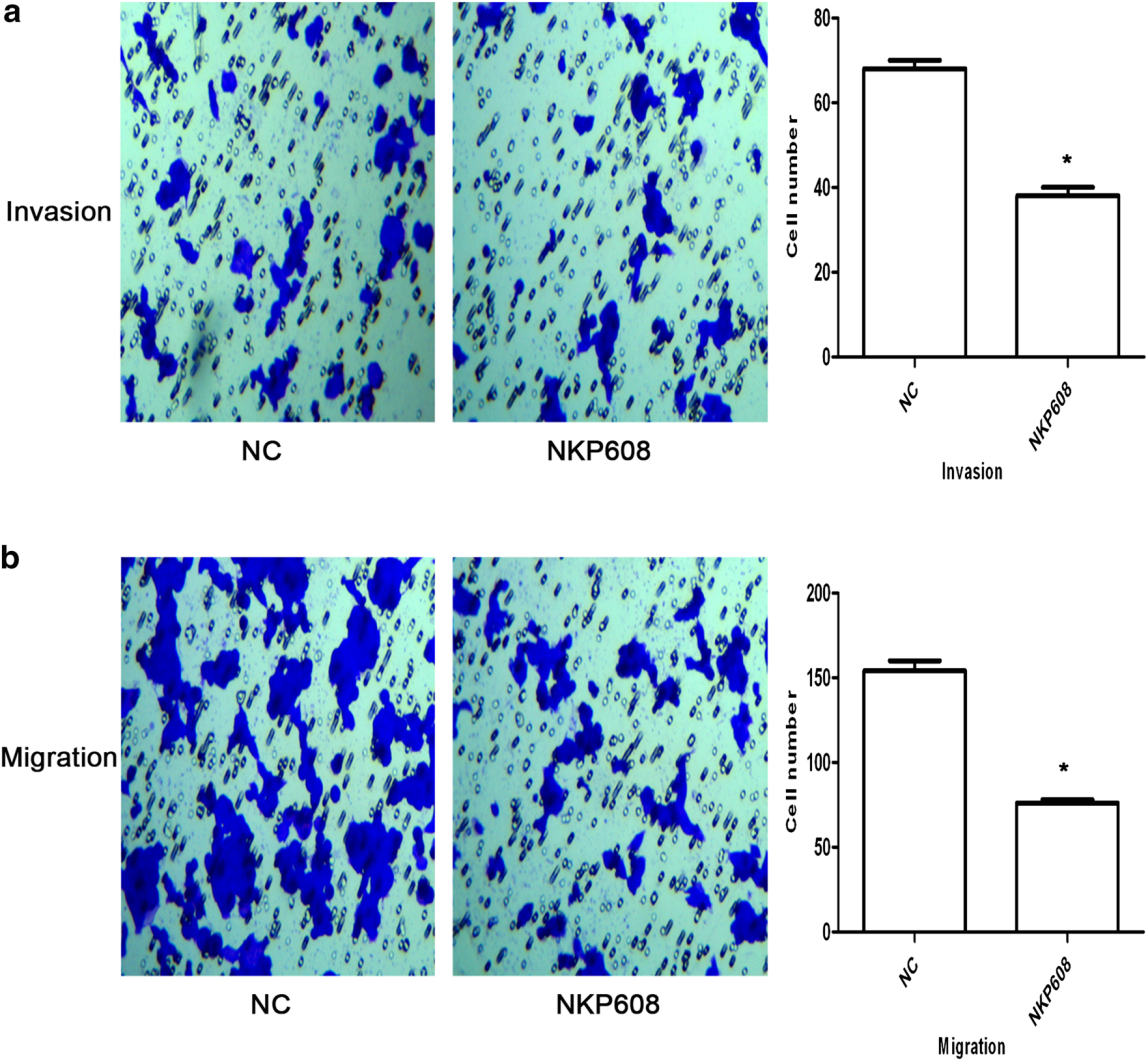
NKP608 attenuated the HCT116 cells migration and invasion ability through transwell chamber invasion/migration assay. **a** Images showing that the invasive cells treated with 24 h incubation of NKP608 were significantly reduced compared with the NC group. **b** Images showing that the migrated cells treated with 24 h incubation of NKP608 were significantly reduced compared with the NC group. Data of the average number of cells were from three independent experiments. *p < 0.05 versus NC group

Next, transwell invasion and migration assay were performed to examine cell invasive and migrated ability. As shown in Fig. 2a, the result of transwell migration assay showed that invasive cell number of NC group was 68 ± 2, while invasive cell number of NKP608 treatment was 38 ± 2 (*p < 0.05). Concomitantly, the result of transwell migration assay demonstrated that the number of cells treated with NC was 154 ± 6, while the number of cells treated with NKP608 was 76 ± 2 (*p < 0.05; Fig. 2b).

Overall, these results demonstrate that NKP608 has a considerably inhibitory effect on the proliferation, invasion and migration of HCT116 cells.

### NKP608 induced apoptosis of HCT116 cells

**Fig. 3:**
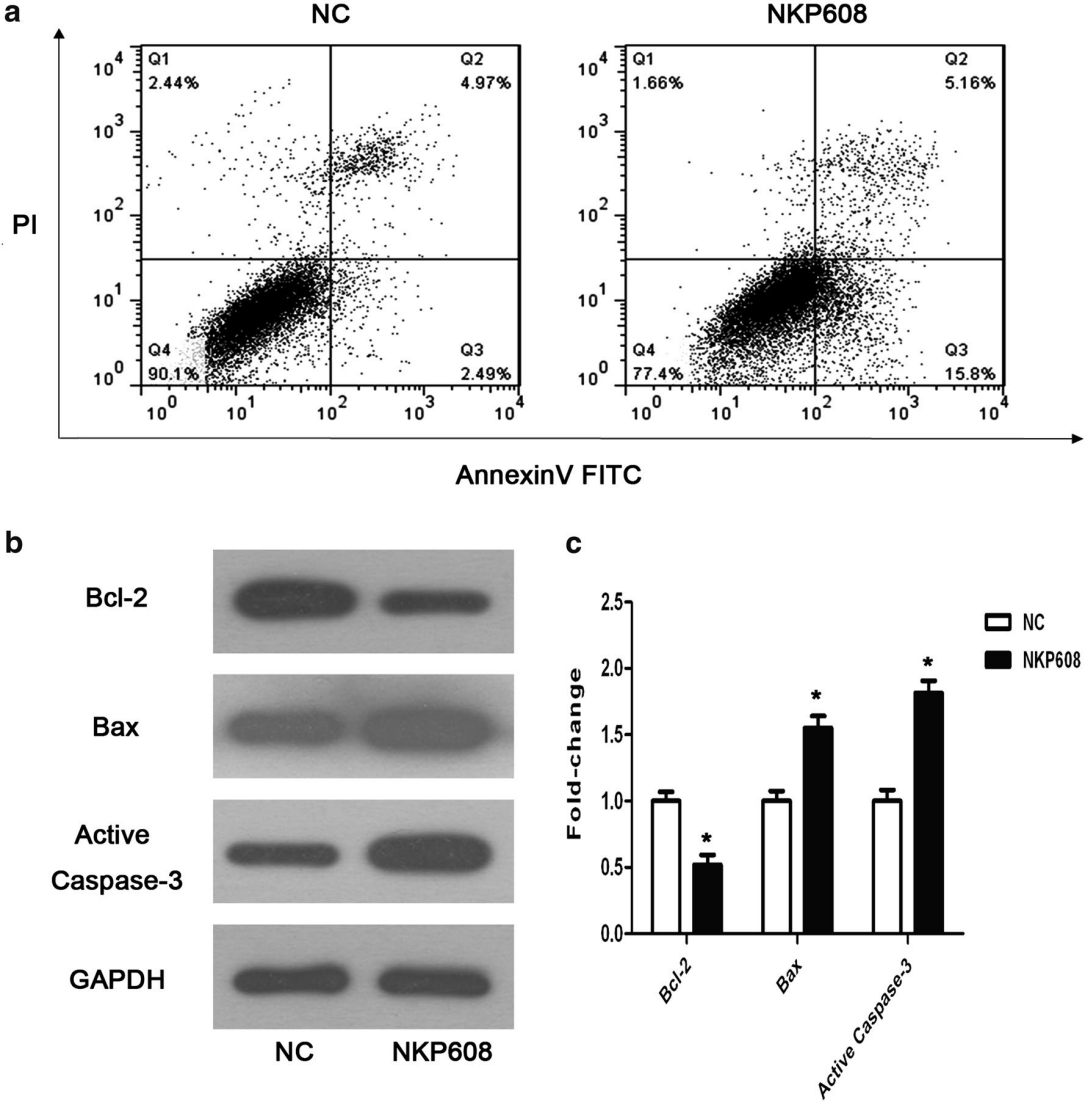
NKP608 causes inhibition of in HCT116 cells. **a** HCT116 cells apoptosis was tested by flow cytometric analysis after Annexin V-FITC/PI staining, and apoptotic cells (Annexin V^+^PI^−^ and Annexin V^+^PI^+^) were shown. **b** Effect of NKP608 on the expression of Bax, Bcl-2 and Caspase-3 protein were detected by western blot assay. **c** Representative western blot images are shown. Values are expressed as the mean ± SD (n = 3). *p < 0.05 versus NC group

To confirm the occurrence of colorectal cancer cells apoptosis upon treatment of NKP608, we assessed apoptosis by Annexin V- FITC/PI staining and flow cytometer analysis. The results indicated that treatment of NKP608 induced markedly apoptotic cell percentage to 20.96 ± 0.73% compared to NC group (7.46 ± 0.34%; Fig. 3a, *p < 0.05). Further, based on the results on the apoptotic rate, we employed western blot assay to detect expression of apoptosis crucially related proteins. As exhibited in Fig. 3b, c, NKP608 obviously promoted the expression of Bax and repressed that of Bcl-2, which was in line with the fact that NKP608 induced apoptosis in HCT116 cells, in addition, the enzymatic activity of Caspase-3 was found to be evidently increased in NKP608-treated cells. Above of these results indicate that NKP608 induce occurrence of colorectal cancer cells apoptosis.

### Effect of NKP608 on Wnt/β-catenin signaling pathway

**Fig. 4:**
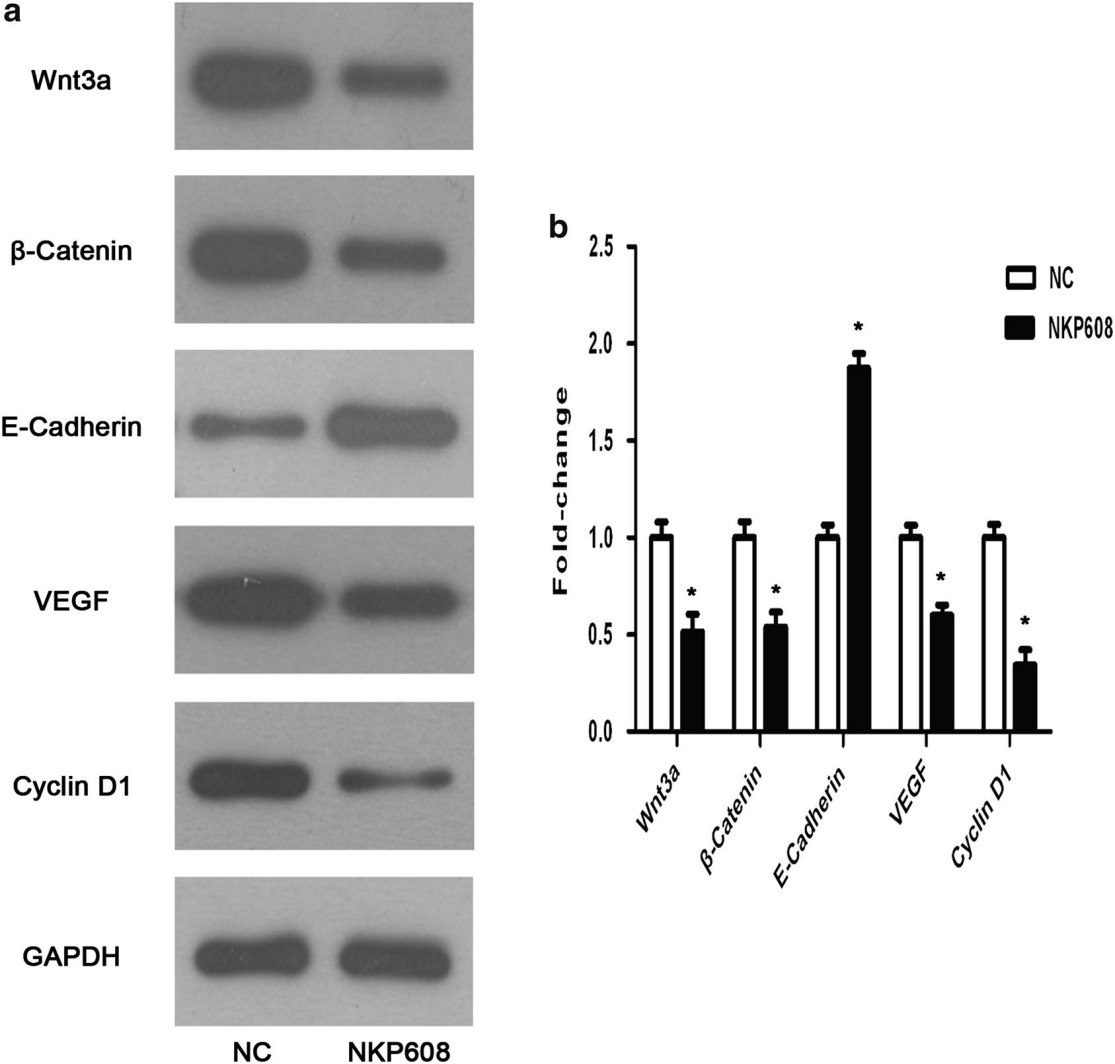
NKP608 inhibited Wnt/β-catenin signaling pathway in colorectal cancer cells. **a** Western blot revealed that NKP608 resulted in an inhibitory action of Wnt relative proteins and proteins relevant cell growth including β-catenin, Wnt-3a, E-Cadherin, Cyclin D1 and VEGF. **b** Quantitative expression levels of proteins are shown. Values are expressed as the mean ± SD (n = 3). *p < 0.05 versus NC group

Wnt/β-catenin signaling is known to be a pivotal pathway closely correlated with carcinogenesis. Thus, to investigate potential mechanism of inhibitory cancer in colorectal cancer cells by NKP608, western blotting was applied to detect Wnt signaling relative members. As shown in Fig. 4, the results revealed that treatment with NKP608 significantly downregulated Wnt-3a and β-catenin, additionally, reduced Wnt downstream target molecules linked with cell proliferation such as Cyclin D1 and VEGF. Meanwhile, western blotting analysis showed that NKP608 increased the expression level of E-Cadherin involved in cellular metastasis and invasion. Overall, these results manifest that NKP608 inhibit colorectal cancer cells growth, invasion and migration might via regulation of Wnt/β-catenin signaling pathway.

## Discussion

The aim of the present investigation was to determine whether NKP608 treatment inhibits cellular proliferation, migration and invasion and induces cellular apoptosis via suppression of the Wnt/β-catenin signaling pathway in HCT116 cells. In addition, NKP608 is first described its antitumor action against colorectal cancer cells and underlying mechanism. Above of reports prompt for the future clinical implications of NKP608 with therapy for colorectal cancer.

Our results in the above demonstrated that NKP608 inhibited cells proliferation, invasion, migration and induced cells apoptosis in HCT116 cell lines, which in agreement with previous works other NK1 receptor antagonist like aprepitant, L-733,060, L-732,138 taking on antitumour activity against diversity of cancerous cells. It is known that the SP/NK1R system enhances the migration and invasion of cancerous cells [13–15], while NK1 receptor antagonists, after binding to NK1 receptor, exerted an anticancer effect [21, 22]. Collectively, these evidence suggested that NKP608 had a potentially inhibitory role in colorectal cancer progression and metastasis.

Despite of NKP608 emerging effects on suppressing colorectal cancer, the potential molecular mechanism by which it regulated demanded further investigation. Herein, we observed NKP608 exhibited an inhibition of Wnt pathway activated, indicated by down-regulation of Wnt-3a and β-catenin. Surely, it is well established that the Wnt signaling involves in controlling cell proliferation, migration, and cell differentiation [23]. Accumulating evidence has suggested Wnt-3a, one of Wnt family members, plays pivotal roles in regulating cell growth via the canonical Wnt signaling pathway in various types of cancer [24–26]. Besides, it is well-known that β-catenin provides a key node in Wnt signaling regulation. Cyclin D1 as a direct transcriptional Wnt signaling target participates in the regulation of cell proliferation and cell cycle progression [19, 27]. Additionally, cell adhesion molecule E-Cadherin acts as an indispensable suppressor of cancer metastasis [28]. Increase of E-Cadherin is able to decrease the transcription of β-catenin, implying that the activity of Wnt/β-catenin signaling was inhibited [29]. To the best of our knowledge, VEGF, as a Wnt target molecular, is involved in cell proliferation, migration and invasion [30, 31]. In line with these findings, in our study, decrease of Wnt-3a, β-catenin, Cyclin D1, and VEGF while an increase of E-Cadherin in NKP608-treated HCT116 cells in comparison with negative groups was observed. Thus, the above findings indicate that NKP608 reduces colorectal cancer cell growth, invasion, migration and induces apoptosis, which was regulated by NKP608 possibly via inhibition of Wnt signaling.

Apoptosis, commonly called programmed cell death, is a prominent hallmark of human cancers [32]. In our investigation, we discovered that anti-apoptotic protein Bcl-2 was downregulated while the levels of pro-apoptotic protein Bax and cleaved Caspase-3 were increased significantly in HCT116 cells after administration of NKP608, which suggested that NKP608, at least partly, induced cell apoptosis in colorectal cancer cells. The enhancement of apoptosis was partly due to the downregulation of Wnt signaling considered as a regulation of cell-growth pathway.

Due to NKP608 beyond 100 µM caused cytotoxic effect on both normal colonic epithelial cell line and colorectal cancer cell lines, NKP608 under 1 µM didn’t work on colorectal cancer cell, so we identified the 10 µM of NKP608 as the effective working concentration. However, this was challenging for retina (2.6 ± 0.4 nM) [33], implying more refined gradient concentration of pharmacological action of NKP608 on colorectal cancer cell needs to explore. Additionally, animal model or further clinical investigation with working concentration remains delicate explorations. The precise mechanism of action of effect of NKP608 on colon cancer is likely to be regulated either through antagonizing NK1 receptor or through further deeper mechanisms. Although further detailed work is needed to fully elucidate the mechanism in-depth of NKP608 in colorectal cancer cells via the Wnt/β-catenin signaling, and even more mechanism or pathways involved, the data of this study may offer valuable insights into the anti-cancer actions of NKP608 and its relation with the Wnt signaling.

A limitation of pharmacological action of NKP608 on colorectal cancer cell was that 10 µM selected as the work concentration in this study.

## Conclusions

In conclusion, the present data indicate that NKP608 has anticancer activities in human colorectal cancer cells through prohibiting the potential of cell proliferation, invasion, migration. Moreover, NKP608 promoted apoptosis of human colorectal cancer cells. What’s more, NKP608 may develop its potential role in suppressing colorectal cancer cells by Wnt inactivation. In light of our findings, NKP608 could be considered as a potential new therapeutic candidate for colorectal cancer.

## Abbreviations

NK1: Neurokinin1
Annexin-FITC/PI: Annexin V-fluorescein isothiocyanate/propidium iodide
VEGF: Vascular endothelial growth factor
SP: Substance P
OD: Optical density
SDS-PAGE: Sodium dodecyl sulfate-PAGE
